# Neutrophil Elastase and Elafin in Inflammatory Bowel Diseases: Urinary Biomarkers Reflecting Intestinal Barrier Dysfunction and Proteolytic Activity

**DOI:** 10.3390/jcm14072466

**Published:** 2025-04-04

**Authors:** Aleksandra Górecka, Katarzyna Komosinska-Vassev

**Affiliations:** Department of Clinical Chemistry and Laboratory Diagnostics, Faculty of Pharmaceutical Sciences in Sosnowiec, Medical University of Silesia in Katowice, 41-200 Sosnowiec, Poland; kvassev@sum.edu.pl

**Keywords:** inflammatory bowel disease, ulcerative colitis, Crohn’s disease, neutrophil elastase, elafin, extracellular matrix, proteolytic balance, biomarker

## Abstract

**Background:** Inflammatory bowel disease (IBD), including ulcerative colitis (UC) and Crohn’s disease (CD), is a chronic inflammatory disorder driven by a complex interplay of immune and proteolytic mechanisms. Neutrophil elastase (NE), released at sites of inflammation, plays a central role by promoting inflammation, degrading the extracellular matrix (ECM), and disturbing intestinal barrier integrity via NF-κB activation and E-cadherin degradation. Elafin, an endogenous NE inhibitor, mitigates proteolytic damage, reinforces the intestinal barrier, and exerts anti-inflammatory effects by suppressing NF-κB and reducing pro-inflammatory cytokines. Since the NE/elafin balance is critical in IBD, assessing their ratio may provide a more precise measure of proteolytic dysregulation. This study aimed to evaluate the diagnostic and prognostic utility of urinary NE, elafin, and their ratio in IBD patients. **Methods:** Urinary concentrations of NE and elafin were measured by immunoassay in 88 subjects including ulcerative colitis and Crohn’s disease patients and healthy individuals. The diagnostic accuracy of these biomarkers was assessed using receiver operating characteristic (ROC) curve analysis. **Results:** Urinary NE levels were significantly elevated in both UC and CD patients compared to controls, with a 17-fold increase in the UC patients and a 28-fold increase in the CD patients (*p* < 0.0001). Elafin levels were also increased in IBD patients. The NE/elafin ratio was significantly increased in both disease groups, with a 4.5-fold increase in the UC and 5.6-fold increase in the CD patients compared to healthy controls. The ROC curve analysis demonstrated that the NE/elafin ratio is the most effective biomarker for distinguishing CD patients from healthy individuals (AUC = 0.896), with a high sensitivity (92.9%) and specificity (69.7%), making it a strong diagnostic tool. NE also showed an excellent diagnostic performance both in CD (AUC = 0.842) and UC (AUC = 0.880). The elafin urinary profile had a high diagnostic value, with a better accuracy in the UC patients (AUC = 0.772) than the CD patients (AUC = 0.674), though it was inferior to NE and NE/elafin. **Conclusions:** Our findings indicate that urinary NE, elafin, the and NE/elafin ratio have significant diagnostic value in differentiating IBD patients from healthy controls. The NE/elafin ratio and NE proved to be the most reliable urinary biomarkers in both CD and UC diagnosis, with a high predictive value and strong discriminatory power.

## 1. Introduction

Inflammatory bowel disease (IBD) is a chronic immune-mediated inflammatory disorder, characterized by alternating episodes of flares and remissions. The pathogenesis of IBD is complex, involving genetic predisposition, gut dysbiosis, loss of intestinal barrier integrity, and an abnormal immune response to microbial or environmental antigens. The two main types of IBD are ulcerative colitis (UC) and Crohn’s disease (CD), which differ significantly in terms of location and extent of lesions, as well as extraintestinal manifestations, possible complications, and the effectiveness of therapeutic approaches. The prevalence of IBD is increasing worldwide as the disease is more commonly diagnosed in patients between the ages of 18 and 35, as well as in the pediatric population. Therefore, considering the rising prevalence and the younger age at diagnosis, early detection is crucial to prevent the development of severe disease and potential complications [1,2,3,4]. At the same time, IBD diagnosis is often delayed due to the heterogeneity of clinical manifestations—especially in Crohn’s disease—and the presence of non-specific symptoms, which may lead to misdiagnosis and further progression of the disease. Reliable and sensitive biomarkers may not only support early IBD diagnosis, but also help to predict its development several years before the onset of clinical symptoms. The diagnosis of IBD is currently based on invasive methods, such as endoscopic examination, while non-invasive ones are mainly limited to measurements of C-reactive protein (CRP) and fecal calprotectin. Elevated fecal calprotectin is observed not only in IBD but also in gastrointestinal infections and colorectal cancer, as well as dietary allergies and celiac disease [1,4]. Therefore, none of the currently studied biomarkers enable IBD diagnosis on their own, let alone effective differentiation between UC and CD. The management of UC and CD requires a more personalized approach and the use of biomarkers that could complement endoscopic examinations in the diagnostic process, as well as facilitate close monitoring of disease progression in order to minimize intestinal damage [5,6,7]. Consequently, there is a pressing need to identify new non-invasive biomarkers that could aid in the diagnosis of IBD and facilitate the differentiation between UC and CD.

The pathogenesis of IBD is related to the increased activation of immune cells, with neutrophils being among the first cells infiltrating the intestinal tissue during disease progression. At the site of inflammation, neutrophils neutralize pathogens through the generation of reactive oxygen species and the release of neutrophil extracellular traps (NETs) containing proteases such as neutrophil elastase (NE).

NE is not only the most abundant serine protease released from neutrophils, but also the most active one, as it accounts for approximately 80% of the proteolytic activity in the human body. The key role of NE is the neutralization of pathogens; however, it has also been suggested to play a role in modulating the inflammatory response by upregulating pro-inflammatory cytokines and thereby enhancing the inflammatory process. NE is also engaged, both directly and indirectly, via the activation of matrix metalloproteinases (MMPs), in the remodeling of the extracellular matrix (ECM) through the cleavage of collagen, laminin, fibronectin, and elastin, thereby contributing to the intestinal damage observed in IBD. Moreover, NE is implicated in another key aspect of IBD pathogenesis, namely the impairment of intestinal barrier integrity, as it can cleave tight junction proteins, such as E-cadherin [8,9,10,11]. Therefore, NE is actively engaged in the development of IBD by amplifying inflammatory processes, damaging intestinal tissue, and impairing intestinal barrier integrity. Considering the multifactorial involvement of NE in IBD pathogenesis, its measurement may be useful not only for the diagnosis of IBD but also as a marker of disease activity. Consequently, in this study the urinary profile of NE will be measured in patients with IBD (both CD and UC) as well as in healthy individuals.

Urine was chosen as the biological material for our study due to its non-invasive collection, allowing for easy and repeated sampling. Additionally, urine can accumulate proteolytic enzymes and their inhibitors, providing insight into the long-term activity of inflammatory processes occurring in IBD. Both NE (≈30 kDa) and elafin (≈6–12 kDa) have relatively small molecular weights, allowing them to freely pass through the glomerular filtration barrier. Unlike serum, where NE is tightly regulated by endogenous inhibitors, especially α1-antytrypsin, urine may better reflect its actual proteolytic activity and imbalances between NE and elafin.

Another biomarker analyzed in our study is elafin, one of the inhibitors of NE. Elafin is a serine protease inhibitor expressed in epithelial cells throughout the entire gastrointestinal tract, as well as in macrophages and neutrophils. Its anti-protease activity is relatively narrow, as it inhibits only neutrophil elastase and neutrophil proteinase 3; however, it can bind to both proteases with equally high affinity. Apart from its elastase inhibitor domain, elafin also possesses transglutaminase substrate domain, which enables its binding to ECM components, thereby protecting ECM from excessive proteolysis. Moreover, this protein demonstrates anti-inflammatory properties as it may inhibit the pro-inflammatory NF-κB pathway, thereby suppressing the inflammatory process [12,13]. During IBD, elafin may not only suppress the proteolytic activity of NE, but also attenuate the inflammatory response. Consequently, a disruption in the NE/elafin balance may lead to intestinal tissue damage, compromised intestinal barrier integrity, and an excessive inflammatory response—features characteristic for IBD. Therefore, in our study, we will evaluate not only the urinary profiles of NE and elafin but also the NE/elafin ratio to elucidate the proteolytic–anti-proteolytic balance in IBD patients. The aim of our study is to assess the utility of NE, elafin, and the NE/elafin urinary profile in the diagnosis of IBD, as well as in differential diagnoses between UC and CD. Additionally, the utility of these biomarkers in evaluating disease activity will also be evaluated.

## 2. Materials and Methods

### 2.1. Study Population

The study included 46 patients with inflammatory bowel disease and 42 healthy individuals. Among the patients with IBD, 30 were diagnosed with ulcerative colitis and 16 with Crohn’s disease. The diagnoses of UC and CD were made at the Department of Gastroenterology of St. Barbara’s Regional Specialist Hospital in Sosnowiec based on clinical symptoms, endoscopic examination, and laboratory tests. Disease activity was evaluated according to the Mayo endoscopic scale in patients with ulcerative colitis and Crohn’s Disease Activity Index (CDAI) in patients with Crohn’s disease. The inclusion criteria for the study group included newly diagnosed active disease and age over 18 years. Patients with unstable coronary disease, bacterial, fungal or viral infections, chronic liver or kidney disease, or toxic or fulminant colitis were excluded from participation in this study. Moreover, pregnant or breastfeeding women were excluded from the study. The inclusion criteria for the control group included age over 18 years and normal results in routine laboratory tests. Exclusion criteria included ongoing pharmacological treatment or/and surgical treatment within 12 months prior to the start of the study. Urine was selected as the biological material investigated in this study. Samples were collected from newly diagnosed patients with UC or CD before treatment, as well as from healthy individuals.

### 2.2. Methods

The concentration of elafin and NE were assessed in urine samples collected from patients with UC and CD, as well as from healthy individuals. Urinary excretion of elafin was evaluated using an ELISA test for elafin from Cloud-Clone Corporation (Houston, TX, USA). The analytical sensitivity of this assay was 0.121 ng/mL, with an intra-assay precision of <10%. Levels of urinary NE were assessed with NE ELISA test from Immunodiagnostik AG Company (Berlin, Germany). The analytical sensitivity of the test used was 0.104 ng/mL, while the intra-assay precision was 6%. To minimize the effect of urine concentration variability, urinary NE and elafin levels were normalized to creatinine levels.

### 2.3. Statistical Analysis

Statistical analysis was performed using STATISTICA software, version 13.3 from StatSoft company (Cracov, Poland). The Shapiro–Wilk test was applied to evaluate the normality of the data distribution, while the significance of differences in urinary levels of elafin and NE between the analyzed groups was evaluated using Student’s *t* test. To further assess the diagnostic utility of the analyzed biomarkers for UC and CD, an analysis of the receiver operating characteristic (ROC) curves was conducted. The correlation between urinary biomarker excretion and both disease activity and C-reactive protein was assessed using the Pearson or Spearman test, depending on the normality of the data distribution. The level of statistical significance was estimated as being lower than 0.05 in the tests performed.

## 3. Results

### 3.1. Characteristic of Patients

Subjects enrolled in this study included 46 patients with IBD (30 UC and 16 CD) and 42 healthy individuals. The clinical characteristics of the IBD patients are presented in Table 1. The UC patient group included 12 females and 18 males, with average age of 33 years old. All of the UC patients had active disease, with a median Mayo endoscopic score of three points. The intensity of the inflammatory response was evaluated using measurements of serum C-reactive protein (CRP) and calprotectin levels. The median CRP level in the UC group was 3.34 mg/L (1.2–15.2), indicating either no or low systemic inflammation. Given that the cut-off value for serum calprotectin in IBD remains under debate, we compared our results with those obtained by Meuwis et al. [14]. In their study, the serum calprotectin level in healthy individuals was 1318 ng/mL, while in the UC group in our study, it was 2782.9 ng/mL. The observed increase in serum calprotectin levels may therefore indicate ongoing gastrointestinal inflammation in patients with UC. Creatinine and sodium levels were within the normal range in all of the patients with UC, although one patient had a decreased potassium concentration and eight individuals had abnormal fasting glucose levels. The group of patients with CD included eight females and eight males, with an average age of 34 years. All of the patients with CD presented with moderately active disease with average CDAI score of 297.73 points. At the same time, the median CRP level was 13.6 mg/L, indicating more intense systemic inflammation in CD patients compared with those with UC. Moreover, serum calprotectin levels were higher in the CD than in the UC patients (3139.5 vs. 2782.9 ng/mL). All of the patients with CD had normal creatinine and potassium levels, whereas two patients were diagnosed with hyponatremia and one had an abnormal fasting glucose level.

### 3.2. Urinary Excretion of Elafin and Neutrophil Elastase in Patients with Ulcerative Colitis and Crohn’s Disease and Healthy Individuals

**Figure 1:**
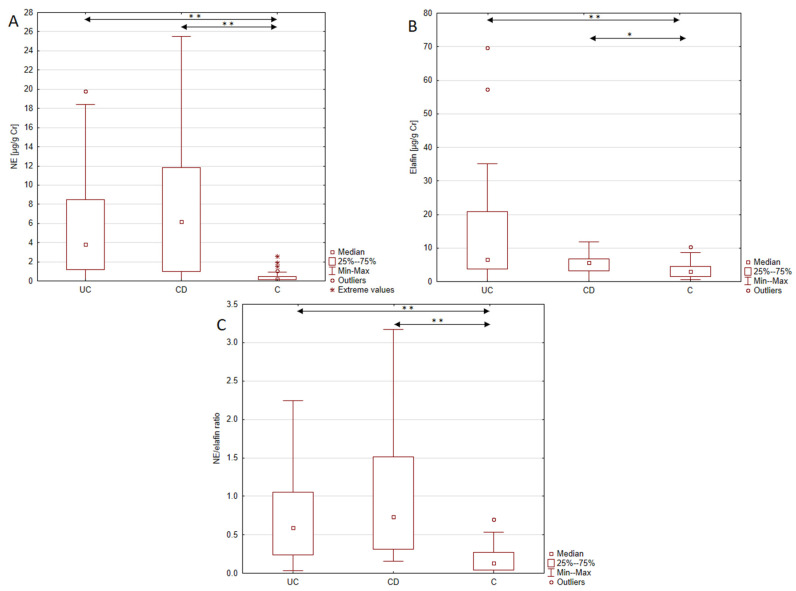
Urinary excretion of elafin and NE and the NE/elafin ratio in patients with ulcerative colitis, and Crohn’s disease and healthy individuals; (**A**), urinary profile of neutrophil elastase in analyzed groups; (**B**), urinary profile of elafin in analyzed groups; (**C**), Neutrophil elastase/elafin ratio in analyzed groups. Data with statistical significance has been marked as * *p* < 0.0005; ** *p* < 0.0001.

In our study, the urinary excretion levels of elafin and NE were measured in patients with UC and CD and healthy individuals, and the results are presented in Table 2 and Figure 1. The mean concentration of elafin was 6.57 μg/g Cr in the UC group, 5.71 μg/g Cr in the CD group, and 3.10 μg/g Cr in the healthy individuals. In the UC group, we observed a statistically significant twofold increase in the elafin level compared to the control group (*p* < 0.0001). A similar nearly twofold increase was also noted in the CD group relative to the control group (*p* < 0.0005). At the same time no significant difference in the elafin profile was observed between patients with UC and CD. Another biomarker analyzed in this study was NE, which presented a significant seventeen-fold increase in the UC group compared to healthy individuals (3.83 vs. 0.22 μg/g Cr, *p* < 0.0001). Moreover, urinary levels of NE were almost 28 times higher in patients with CD compared to healthy individuals (6.17 vs. 0.22 μg/g Cr; *p* < 0.0001). Given that elafin is an inhibitor of NE, we also evaluated the NE/elafin ratio in both the study and control groups. Patients with both UC and CD presented significantly increased NE/elafin ratio compared to healthy individuals. Among UC patients, the observed increase reached 4.5-fold, while CD patients presented a 5.6-fold increase in the NE/elafin ratio compared to healthy individuals. At the same time, no significant difference was noted between the UC and CD groups.

### 3.3. Urinary Elafin and Neutrophil Elastase as Biomarkers of Ulcerative Colitis and Crohn’s Disease

**Figure 2:**
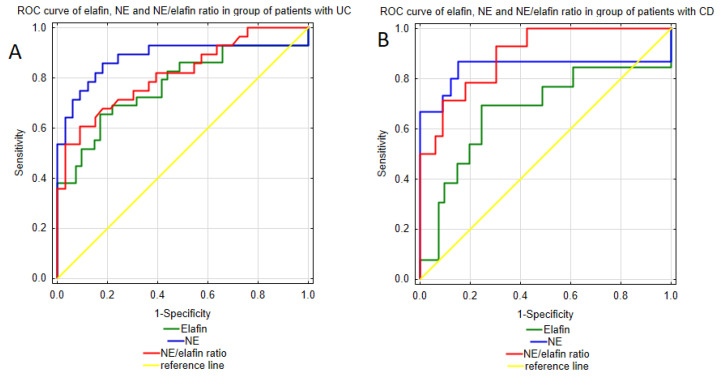
ROC curve of NE, elafin, and NE/elafin ratio as diagnostic biomarkers of UC and CD; (**A**), ROC curve of analyzed parameters as diagnostic biomarkers of UC group; (**B**), ROC curve of analyzed parameters as diagnostic biomarkers of UC group.

The aim of our study is to evaluate the role of urinary elafin and neutrophil elastase, along with the NE/elafin ratio, in the diagnosis and monitoring of ulcerative colitis and Crohn’s disease. Therefore, to assess the diagnostic utility of these biomarkers we performed ROC curve analysis of the urinary profile of NE and elafin, as well as the NE/elafin ratio, with the results presented in Table 3 and Figure 2. In this study, higher diagnostic performance values were noted for the urinary NE/elafin ratio profile in CD patients. This biomarker demonstrated excellent accuracy in distinguishing CD patients from healthy individuals with an AUC of 0.896 (0.805–0.988). Moreover, the test exhibited 92.9% sensitivity, 69.7% specificity, a 56.5% PPV, and a 95.8% NPV, resulting in great recognition of CD patients, but with a simultaneous risk of CD overdiagnosis. The diagnostic performance of NE/elafin ratio was also promising in the group of patients with UC. ROC curve analysis revealed a strong ability of this biomarker to differentiate UC patients from healthy individuals, with an AUC of 0.815 (0.708–0.923). This test also showed high values of specificity (90.9%), PPV (85%) and NPV (73.2%), however lower value of sensitivity (60.7%), which may indicate its greater potential in excluding UC.

The urinary NE profile demonstrated strong performance in distinguishing CD patients from healthy individuals, with an AUC of 0.842 (0.672–1), as well as high sensitivity (86.7%) and specificity (84.8%). When analyzing the PPV and NPV (72.2% vs. 93.3%), however, this test exhibited greater reliability of negative test result. In group of patients with UC, the urinary NE profile also demonstrated excellent diagnostic performance effectively differentiating patients with UC from healthy individuals, with an AUC of 0.880 (0.778–0.981). Given the high values of sensitivity (85.7%) and specificity (81.8%), urinary NE measurements may be useful for accurate UC diagnosis. At the same time, the PPV and NPV were high, indicating the strong predictive value of both positive and negative test results.

Moreover, in this study, we assessed the diagnostic utility of elafin profile in both UC and CD. The elafin measurements presented strong discriminative ability in distinguishing patients with ulcerative colitis from healthy individuals, with an AUC of 0.772 (0.653–0.891), high specificity (82.9%) and satisfactory sensitivity (65.5%). Additionally, the PPV equaled 73.1% and the NPV was 77.3%, indicating strong predictive value of both positive and negative test results. In group of patients with CD, diagnostic indicators were slightly lower. ROC curve analysis presented moderate ability of elafin assessments in differentiating patients with CD from healthy individuals with an AUC of 0.674 (0.480–0.867). Furthermore, the test had a sensitivity of 69.2% and specificity of 75.6%, indicating a similar ability to correctly identify both CD and healthy individuals within the population. However, the positive test results presented a lower PPV, indicating possible overdiagnosis of CD.

### 3.4. Utility of Urinary Biomarkers in Disease Activity Monitoring

In this study, apart from the diagnostic utility of the analyzed biomarkers, we also evaluated their usefulness in monitoring the disease activity. Statistical analyses revealed no significant correlation between the urinary profiles of elafin, NE, and the NE/elafin ratio with disease activity in either the UC and CD group. Similarly, no significant relationship was observed between the analyzed biomarkers and the CRP level in both study groups.

## 4. Discussion

The identification of reliable biomarkers for IBD is crucial for early and accurate diagnosis, which not only guides treatment strategies but also helps to predict the disease course. The accurate and early identification of UC or CD using reliable biomarkers may not only limit the extent of intestinal tissue injury, but also allows for prompt initiation of therapy, thereby increasing the likelihood of achieving clinical remission within the first few months of treatment [6]. Although fecal calprotectin has been identified as a diagnostic biomarker of IBD, its clinical utility remains restricted due to its lack of specificity in distinguishing IBD from other inflammatory gastrointestinal disorders. Therefore, in our research we aimed to identify new biomarkers that could support the diagnostic process for IBD and aid in differentiating UC from CD. The biomarkers assessed in this study (NE, elafin, NE/elafin ratio) were selected based on their active role in key processes related to the pathogenesis of IBD, including intestinal barrier disruption, excessive immune response, and intestinal tissue damage. Urine was chosen as the biological material for analysis due to its accessibility, non-invasive collection, and ability to reflect biochemical changes that occur during disease progression. In serum, NE activity is strictly regulated by endogenous inhibitors such as α1-antitrypsin (α1AT). However, in urine, the NE-α1-antitrypsin complex is typically absent or present only in trace amounts due to the high molecular weight of α1AT. Therefore, urine may provide a more accurate representation of NE’s actual proteolytic activity while also reflecting the balance between NE and elafin, as both have low molecular weights.

To our knowledge, this is the first study demonstrating increased urinary excretion of NE in patients with IBD (both UC and CD) compared to healthy individuals. The observed increase in NE excretion reached 17-fold in UC and 28-fold in CD patients compared to the control group. The demonstrated upregulation of NE is in line with the results of Kuno et al. [15], who noted elevated expression of NE in mucosal biopsies from UC patients compared to controls. Researchers have also noted differences in NE levels between inflamed and non-inflamed tissues from UC patients, while no such difference was observed between non-inflamed UC tissue and control samples. Moreover, the level of NE correlated positively with the number of neutrophils and mononuclear cells, indicating a local inflammation-driven increase in NE during UC. Additionally, in a study conducted by Curciarello et al. [16], the NE activity in the mucosal tissue of IBD patients was elevated and increased over time, whereas it remained stable in the control group, suggesting both enhanced and prolonged NE activity in UC patients. NE also plays a role in ECM proteolysis, releasing pro-inflammatory cytokines deposited within the matrix and modulating the inflammatory response via Toll-like receptor 4 and the NF-κB pathway, thereby contributing to the development of inflammatory processes. Ginzberg et al. [17] demonstrated in a cellular model of the intestinal epithelium that NE migration across the epithelial barrier was associated with E-cadherin degradation, detachment of the epithelial monolayer, and the disruption of adherens junction integrity. These actions of NE may lead to increased intestinal and ulcer formation—key features of IBD. Additionally, some researchers indicate that NE may limit epithelial proliferation and induce epithelial-to-mesenchymal transition, resulting in impaired mucosal repair. Taken together, these findings highlight NE as a protease with a substantial contribution to intestinal tissue damage progression. This causal role of NE in the development of UC may explain its increased urinary excretion in UC patients [9,15,18]. In contrast, reports regarding NE expression in CD patients remain inconsistent. In our study, NE excretion in CD patients was significantly higher than in healthy individuals. However, in Kuno et al.’s [15] study, NE expression did not differ between CD patients and controls. The observed inconsistency may be related to differences in treatment and disease duration, as our study included only newly diagnosed, treatment-naïve patients, whereas the study by Kuno et al. involved CD patients diagnosed 15 months to 18 years prior to the study, many of whom received treatment with 5-amino salicylic acid or prednisolone. Conversely, Langhorst et al. [19] reported an increased expression of fecal NE in both CD and UC patients compared to patients with irritable bowel syndrome (IBS), identifying NE as a superior marker for IBD compared to CRP. Moreover, measurements of fecal NE allow active IBD to be differentiated from inactive IBD, indicating its potential role in monitoring the disease activity. Among the analyzed parameters, neutrophil elastase emerged as a promising biomarker for IBD diagnosis. Conducted ROC curve analysis demonstrated excellent discriminative ability of urinary NE in distinguishing Crohn’s disease patients and healthy individuals. Moreover, the analysis revealed high sensitivity and specificity of NE urinary profile, supporting its potential as a diagnostic biomarker for CD. Considering the higher NPV compared to the PPV (93.32% vs. 72.2%), this test may overdiagnose CD. The ROC curve analysis of urinary NE in UC patients also yielded encouraging results, demonstrating a strong ability to differentiate patients with UC from healthy individuals with high specificity and sensitivity. These findings, together with the strong values of both the positive and negative test results, highlight the potential clinical application of urinary NE measurements in the diagnosis of UC.

Furthermore, this study demonstrated increased urinary excretion of elafin in both UC and CD patients compared to the control group. The results obtained are in line with results of Wang et al.’s [20] study, who noted increased serum elafin levels in IBD patients. Similar results were presented in Krawiec et al.’s [21] study, which was conducted in a pediatric IBD population. The up-regulation of elafin in IBD was also presented in a study by Schmid et al. [22], which reported increased elafin expression in inflamed intestinal tissue compared to non-inflamed tissue from IBD patients. The increase in elafin levels observed in IBD may be related to the inflammatory process, as this protein is constitutively expressed in intestinal epithelial cells, but its expression increases in response to inflammatory stimuli. Key triggers of elafin up-regulation include the IL-1β and TNF-α-two cytokines, which are known to play a pivotal role in IBD pathogenesis [23,24]. Beyond its role as an NE inhibitor, elafin is involved in maintaining intestinal barrier integrity and exerts anti-inflammatory effects. This protective function of elafin was demonstrated in the study conducted by Motta et al. [24] using both cellular and animal models of IBD. In that study, elafin not only inhibited TNFα-induced intestinal barrier permeability, but also improved the organization of tight junction proteins. Moreover, elafin administration suppressed the inflammatory response by reducing the levels of IBD-related pro-inflammatory cytokines including IL-6, IL-8, IL-17, TNF-α, as well as down-regulating the NF-κB pathway [24,25,26]. These findings suggest that despite its protective role against colitis and its up-regulated expression in IBD, the effect of elafin may not be sufficient to suppress the pro-inflammatory processes underlying IBD. Nevertheless, given its involvement in the key pathological mechanisms of IBD—namely intestinal barrier integrity and inflammatory process—we assessed the diagnostic utility of elafin measurements in IBD. The urinary elafin profile proved useful in diagnosing both UC and CD, with a better diagnostic performance in UC patients. Urinary elafin measurements effectively distinguished UC patients from healthy individuals with high specificity and satisfactory sensitivity. Moreover, the urinary elafin measurements demonstrated a strong PPV and NPV, indicating a low incidence of false positive and false negative diagnoses. In CD patients, the elafin urinary profile also showed a strong ability to differentiate CD patients from healthy individuals with a slightly better sensitivity but a lower specificity, suggesting a higher susceptibility to false positive diagnoses. Additionally, in CD patients, urinary elafin levels exhibited a lower variability, suggesting greater stability of this biomarker in CD patients.

In this study, the increase in NE levels was accompanied by an elevation of elafin expression; however, this up-regulation of elafin may not counteract NE activity in IBD. Consequently, the protease–anti-protease balance may remain disrupted, contributing to intestinal tissue damage. These findings are supported by the study of Curciarello et al. [16], which assessed the protease–anti-protease balance in the mucosal tissue of IBD patients and healthy individuals. Despite increased elafin expression, patients with IBD exhibited enhanced NE activity, which was further reflected by a decreased elastin level, an extracellular matrix component degraded by NE. To exclude a reduced suppressive effect of elafin on NE, the researchers evaluated NE’s sensitivity to elafin-mediated inhibition. Upon exposure to elafin, elastin proteolysis was diminished, confirming that NE remained responsive to elafin’s inhibitory effects. These findings emphasize that measurements of NE and elafin concentrations alone may be insufficient to assess the proteolytic–anti-proteolytic balance during IBD. Assessments of the NE/elafin ratio may provide a more comprehensive reflection of the protease—anti-protease imbalance, which could directly contribute to the degradation of the extracellular matrix and subsequent damage of intestinal tissue observed in both UC and CD. Moreover, in a previously mentioned study by Curciarello et al. [16], an imbalance between NE and elafin was related to a loss of response to biological treatment in UC patients, likely due to NE’s ability to neutralize anti-TNF-α agents. At the same time, this effect was mitigated by the administration of exogenous elafin. These findings suggest that the disruption of the NE/elafin balance may not only contribute to disease pathogenesis, but also to a loss of response to anti-inflammatory biological treatment in IBD patients. Therefore, in this study we additionally assessed the NE/elafin ratio to better illustrate the proteolytic–anti-proteolytic balance in UC and CD patients compared to healthy individuals. The results revealed a 4.5-fold increase in the NE/elafin ratio in patients with UC and a 5.6-fold increase in CD patients compared to healthy individuals, indicating an imbalance in proteolytic and anti-proteolytic activity in IBD. These findings correspond with the results obtained in the studies conducted by Motta et al. [25], Barry et al. [9], and Schmid et al. [21], which also demonstrated a disrupted proteolytic–anti-proteolytic balance during IBD and highlighted its causative role in intestinal tissue damage. Moreover, enhanced NE activity in IBD may not only drive inflammation but also contribute to abdominal pain. NE has been shown to cleave protease-activated receptor 2 (PAR2), leading to the sensitization of nociceptive neurons and subsequent development of neurogenic inflammation and pain [27,28]. Despite the recognized role of the protease–anti-protease balance in IBD pathogenesis, the NE/elafin ratio has not yet been considered as a potential diagnostic biomarker. Therefore, in this study, we assessed the NE/elafin excretion ratio to explore its potential utility in diagnosing UC and CD. Among the analyzed biomarkers, the NE/elafin ratio demonstrated the best potential in distinguishing patients with Crohn’s disease from healthy individuals, with a 92.9% of sensitivity, indicating a low risk of CD misdiagnosis. Similarly, in UC patients, the NE/elafin ratio effectively distinguished UC patients from healthy individuals, with 90.9% specificity and 60.7% sensitivity. These results suggest that the assessment of the NE/elafin ratio in UC patients is more prone to omit UC patients in population. Comparable results were observed considering NE urine profile, which biomarker effectively distinguished both UC and CD patients with high sensitivity and specificity. Regarding the AUC values of the analyzed biomarkers, the NE/elafin ratio and NE provided better diagnostic values compared to elafin measurements in urine. Given the potential role of NE/elafin as an indicator of treatment response—as suggested by Curciarello et al. [16]—measurements of this ratio might prove useful in monitoring therapeutic efficacy during biological treatment. Since the study cohort consisted exclusively of newly diagnosed patients who had not yet received biological treatment, the potential of this ratio as a treatment monitoring tool should be further analyzed in future studies.

The biomarkers—elafin, NE, and the NE/elafin ratio—evaluated in this study demonstrated potential for the early detection of both UC and CD, which could ultimately contribute to improved clinical outcomes through earlier intervention. A key strength of our study lies in the inclusion of only newly diagnosed UC and CD patients, allowing us to assess the diagnostic value of selected biomarkers. Additionally, the use of urine samples offers the advantage of a non-invasive, easily repeatable method of biomarker assessment. Moreover, due to the high molecular weight of α1-antitrypsin, its filtration into urine is significantly limited, which restricts the formation of NE-α1–antitrypsin complexes in urinary samples. As a result, urinary measurements of free NE may more accurately reflect its true proteolytic potential and the imbalance between NE and its physiological inhibitor, elafin. In this study, NE and elafin concentrations were normalized to the urinary creatinine level in each subject to minimize the effect of urine concentration variability. This standardization allows us to assess each biomarker in a single urine sample, reducing the need for 24 h urine collection. At the same time, referencing the urinary creatinine level helps to decrease the error related to intra- and inter-individual variability in the NE and elafin profiles among tested subjects. Nonetheless, this study has some limitations, among which the relatively small size of study groups may limit the ability to generalize the obtained results to a larger population of IBD patients. Consequently, the findings reported here should be interpreted with caution and validated in a larger independent cohort of patients with different disease manifestations, especially in the case of patients with Crohn’s disease, to ensure greater variability in and the external validity of the analyzed biomarkers. Additionally, external validation would allow for the assessment of both intra-individual and inter-individual variability, which may influence the accuracy and reproducibility of urinary elafin and NE measurements in IBD patients.

## 5. Conclusions

In summary, all analyzed biomarkers—neutrophil elastase, elafin and the NE/elafin ratio-demonstrated significant potential for the diagnosis of both ulcerative colitis and Crohn’s disease. Among them, the NE/elafin ratio emerged as the most promising biomarker, exhibiting a high sensitivity and specificity in distinguishing both UC and CD patients from healthy individuals, with a particularly greater accuracy in the CD group. Moreover, NE presented a comparable capability to differentiate both UC and CD patients from healthy subjects with high values of diagnostic indicators. Elafin presented a very good ability to diagnose IBD patients with a superior diagnostic performance in UC patients compared to CD patients, indicating a potentially greater diagnostic value in UC patients. Taken together, the biomarkers evaluated in this study may serve as supportive non-invasive tools in the diagnostic workup of UC and CD; however, further studies on larger cohorts are warranted to validate these findings.

## Figures and Tables

**Table 1:** Clinical characteristics of patients.

Parameter	UC	CD
Number of patients	30	16
Sex [female/male]	12/18	8/8
Age [years]	33 ± 13	34 ± 9
Body Mass Index [kg/m^2^]	24.3 ± 3.6	20.6 ± 3.4
Disease activity	Mayo endoscopic scale: 3 (2–3)	CDAI: 297.73 ± 38.88
C-reactive protein [mg/L]	3.34 (1.26–15.2)	13.6 (3.2–24.4)
Serum calprotectin [ng/mL]	2782.9 (1674.2–4754.5)	3139.5 ± 1361.6
Creatinine [mg/dL]	0.88 ± 0.15	0.91 ± 0.17
Sodium [mmol/L]	140 ± 1.89	138.06 ± 2.86
Potassium [mmol/L]	4.16 ± 0.41	4.27 ± 0.27
Glucose [mg/dL]	89.59 ± 12.80	86.70 ± 8.30

Data are presented as mean ± standard deviation in case of normally distributed data and median and interquartile range in non-normally distributed data.

**Table 2:** Urinary excretion of elafin and NE in patients with ulcerative colitis and Crohn’s disease and healthy individuals.

Parameter	UC	CD	C	*p* UC vs. C	*p* CD vs. C	*p* UC vs. CD
elafin [μg/g Cr]	6.57 (3.69–20.88)	5.71 (3.12–6.80)	3.10 (1.46–4.59)	<0.0001	<0.0005	>0.05
NE [μg/g Cr]	3.83 (1.18–8.50)	6.17 (0.97–11.84)	0.22 (0.13–0.45)	<0.0001	<0.0001	>0.05
NE/Elafin ratio	0.59 (0.24–1.05)	0.73 (0.31–1.51)	0.13 (0.04–0.27)	<0.0001	<0.0001	>0.05

Results have been presented as median with interquartile range. C, healthy control; CD, patients with Crohn’s disease; NE, neutrophil elastase; UC, patients with ulcerative colitis; μg/g Cr, μg/g creatinine.

**Table 3:** ROC curve analysis of the urinary profile of the analyzed biomarkers.

Parameter	Analyzed Groups	AUC	Youden Index	Cut-Off	Sensitivity	Specificity	PPV	NPV
elafin	UC vs. C	0.772 (0.653–0.891)	0.48	5.89 μg/g Cr	65.5%	82.9%	73.1%	77.3%
	CD vs. C	0.674 (0.480–0.867)	0.45	4.92 μg/g Cr	69.2%	75.6%	47.3%	88.6%
NE	UC vs. C	0.880 (0.778–0.981)	0.68	0.76 μg/g Cr	85.7%	81.8%	80%	87.1%
	CD vs. C	0.842 (0.672–1)	0.72	0.82 μg/g Cr	86.7%	84.8%	72.2%	93.3%
NE/Elafin ratio	UC vs. C	0.815 (0.708–0.923)	0.52	0.40	60.7%	90.9%	85.0%	73.2%
	CD vs. C	0.896 (0.805–0.988)	0.63	0.25	92.9%	69.7%	56.5%	95.8%

AUC, area under the ROC curve; CD, Crohn’s disease; NE, neutrophil elastase; NPV, negative predictive values; PPV, positive predictive value; UC, ulcerative colitis; μg/g Cr, μg/g creatinine.

## Data Availability

Data are available in the manuscript.
